# Analysis of the possibilities of reducing the levels of residual stresses in casting produced from synthetic cast iron

**DOI:** 10.1016/j.heliyon.2024.e33623

**Published:** 2024-06-25

**Authors:** Peter Futas, Miroslav Pástor, Alena Pribulova

**Affiliations:** a Faculty of Materials, Metallurgy and Recycling, Technical University of Kosice, Letna 9, 042 00, Kosice, Slovak Republic; b Faculty of Mechanical Engineering, Technical University of Kosice, Letna 9, 042 00, Kosice, Slovak Republic

**Keywords:** Failure analysis, Residual stresses, Mechanical properties, Casting, Synthetic cast iron

## Abstract

In the production of castings, residual stresses arise in the cooling process, the level of which is often unknown. Their significance in engineering practice is very important because they are superposed on the stresses from the service load and are often the primary cause of material failure leading to failure of the equipment or structure. Their quantification using numerical simulations is rather difficult because many variables enter into the calculation simulating technological processes. Therefore, residual stress levels are most often determined in such cases by experimental measurement and, if possible, by monitoring and evaluating the history of parameter changes due to changes in the input parameters.

In the present paper, the results of experimental measurements of residual stresses in synthetic cast iron castings are presented, where the effect of Ti microalloying on residual stress levels was assessed. Based on the comparison of the results obtained experimentally on castings made from grey cast iron, it can be concluded that the addition of Ti metal reduced the residual stress levels while maintaining the tensile strength and hardness HB.

## 1 Introduction

Cast iron is the most suitable material for the production of thermally stressed castings that are heated or cyclically thermally stressed for long periods. These are mainly metallurgical castings (die casting mold, furnace fittings, crucibles, grates, pipes, etc.), but also castings for the automotive industry and households. To reduce the cost of producing cast iron castings, pig iron is replaced by cheaper and more affordable steel scrap. Cast iron made from steel scrap is called synthetic cast iron. It has been known for more than 60 years and its production was made possible by the development of crucible electric induction furnaces (EIF). Their advantage lies mainly in the benefit of producing liquid metal with a precise chemical composition using up to 100 % of the steel scrap in the charge [[1], [2], [3]]. Carburizing agents with low sulphur, nitrogen, ash, and volatile matter content must be used to produce such cast iron. The high proportion of steel scrap in the charge increases the risk of undercooling. It is therefore necessary to control and monitor the production process using cooling curves [4]. Contaminated steel scrap increases the hydrogen content of the liquid metal, the loss of metal by burnout, and the amount of slag. Therefore, it is very important to sort steel scrap for alloying elements (Cr, Mn, Ni, Cu) and unwanted metals (Cu, Pb, Sn, Zn) that enter the liquid metal and can lead to their increased content in the cast iron [[5], [6], [7]].

Already at the beginning of the production of synthetic cast iron, it was found to have a higher HB hardness at the same degree of saturation (Sc) compared to cast iron produced from pig iron. The high hardness of HB causes problems in the final machining of the castings. Synthetic cast iron also has the disadvantage of increasing stresses in castings. These negative properties are attributed to the nitrogen content, which is introduced into the melt by increasing the proportion of scrap steel in the casting. The effect of nitrogen can be neutralized by the addition of Ti metal or FeTi and TiO_2_ oxides (FeTiO_3_) [[8], [9], [10], [11]].

The solidification of the melt in the mold as a result of its cooling produces a casting of the desired shape and dimensions, with the result that the melt becomes powdery, that means its dimensions are reduced. At the same time, residual stresses, referred to as internal (endogenous) stresses, are generated in it. Caused by Ref. [12]:

- uneven cooling of individual parts of the casting, i.e. different temperatures in individual parts of the casting - thermal stresses (endogenous),
- resistance of the mold (part of it) against casting shrinkage - shrinkage stress (exogenous),
- volume changes during phase transformations - phase or transformation voltage.

Residual stresses exist in the equilibrium state inside the castings without the action of external forces, mainly due to non-uniform plastic deformation. Stresses in different parts of the casting are induced by both external (exogenous) and internal (endogenous) forces that counteract the free expansion of the casting when it is cooled or heated (heat treatment or cyclic thermal stresses. A negative consequence of internal stresses is the development of casting defects such as shape distortions, dimensional changes, continuity failures - cracks and fissures, and residual internal stresses [[13], [14], [15], [16]].

To ensure long-term durability and, in particular, to reduce the consumption of thermally stressed castings, it is important to have an optimum design to ensure that minimum thermal differences and, therefore, stresses are generated. This is a difficult problem for design engineers, which is solved by numerical simulation. In practice, several techniques are used to predict the occurrence and magnitude of residual stresses [17,18]. Their common feature is the experimental verification of their correctness [19].

This paper presents the quantification of residual stresses on castings from 3 different melts. The effects of melt parameters on residual stress levels were proved by experimental measurements.

## 2 Materials and methods

Experimental measurements of residual stresses were carried out on three samples cast from grey cast iron EN GJL-250 (degree of saturation Sc 0.87–0.93; C content 3.1–3.3 %; Si content 1.7–1.9 %) in the operating conditions of the Slovak foundry, which has electric induction furnaces (Otto Junker with a capacity of 2 × 6 tons). The share of steel scrap in the charge was changed in the realized melts and its impact on the foundry and technological properties of the cast iron was eliminated by metallurgical interventions. A total of 6 experimental melts were designed and realized, in which the influence of the increased content of steel scrap in the charge and the elimination of its negative effects on the final quality of the cast iron were studied:

a) by increasing the overheating temperature (1500 °C, normally 1420 °C) and by inoculation,
b) by alloying with titanium (overheating to 1500 °C and inoculation),
c) by increasing the C content (+0.5 %) with reduced Si content.

**Fig. 1:**
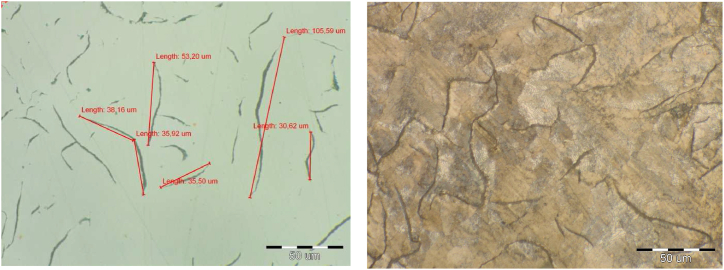
Microstructure of melt No. 1.
- Tensile strength (Rm) comparison – performed on test bars with diameter of 30 mm.

Titanium was added as FeTi70 to the furnace with the charge. Inoculation is two-stage, half of the FeSi75 inoculant is added to the furnace and the other part to the iron stream during casting. The cast iron from the experimental melts were compared with commonly produced semi-synthetic cast iron (the portion of steel scrap in the charge is about 33 %). From each melt were cast and evaluated:

- Chemical analysis,
- R-block (Stairs test with a three thicknesses: 100, 50 and 20 mm – standard thickness of the cast wall, Fig. 1), to determine the sensitivity difference between the wall thickness and the cast hardness (cooling rate),

Castings were gravity cast into the bentonite molding mixture, on a semi-automatic or automatic line. The test molds were prepared at the same time as the molds for the brake disc castings. The samples were cast under the same conditions as the castings, the cast temperatures were 1380–1400 °C.

The results from the metallurgical analysis and mechanical properties (tensile strength TS and hardness HB) have been continuously published in the literature and their results are summarized in Table 1 and Table 2 [20].

**Table 1:** The portion of the charge material and overheating temperature [20].

Melt No.	Charge material (wt. %)								Temperature [°C]
	Steel scrap (sheet metal)	Return material	PIG iron	FeSi75	FeMn80	Carburizer (Desulco 9001)	Inoculant (added to the iron stream)	FeTi70	
1.	32.8	53.3	10	–	0.4	1.3	0.20	–	1420
2.	35.5	52.9	9.8	0.12	0.5	0.98	0.20	–	1500
3.	35.5	52.9	9.8	0.12	0.5	0.98	0.20	0.28^a^	1500
4.	82.7	–	13	0.22	0.47	3.45	0.20	–	1450
5.	97.8	–	–	0.59	0.4	0.67	0.34 + 0.2^b^	–	1450
6.	97.5	–	–	0.60	0.47	0.67	0.50	0.26^a^	1520

a FeTi60 - into the furnace.

b Pre-inoculation (SiC) - into the furnace.

**Table 2:** Chemical composition, Sc, CE and measured mechanical properties [20].

Melt No.	wt. [%]							Sc	CE [%]	TS [MPa]	HB
	C	Si	Mn	P	S	Ti	N_2_				
1.	3.23	1.612	0.657	0.024	0.025	–	0.0113	0.848	3.721	297	186 a(205)
2.	3.33	1.52	0.664	0.024	0.021	–	0.0113	0.868	3.793	352	204 a(223)
3.	3.32	1.486	0.658	0.023	0.021	0.183	0.0091	0.863	3.773	268	217 a(224)
4.	3.79	1.026	0.773	0.018	0.012	–	0.0073	0.951	4.103	256	211 a(221)
5.	3.28	1.69	0.84	0.06	0.016	–	0.0175	0.869	3.805	258	243 a(312)
6.	3.13	1.61	0.79	0.016	0.011	0.192	0.0205	0.821	3.618	220	197 a(209)

a hardness HB measured on a thin section R-block (20 mm).

The samples for metallographic analysis were prepared in the form of test bars and prepared traditionally.

R-Block was cut in each of the cross-sectional areas. For every section, the Brinell hardness was measured. The hardness was measured using the HPO 3000 durometer (setting: 10/3000/10), which means that the diameter of the testing ball was set to 10 mm, and the strength was set to 3000 N for a duration period of 10 s.

Tensile strength was measured on test bars (Ø30 mm) on a standard testing machine of the ZWICK brand.

**Fig. 2:**
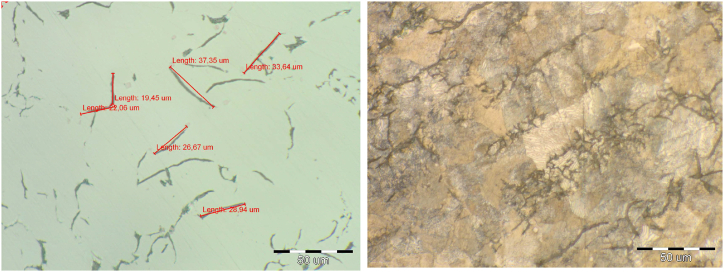
Microstructure of melt No. 3.

**Fig. 3:**
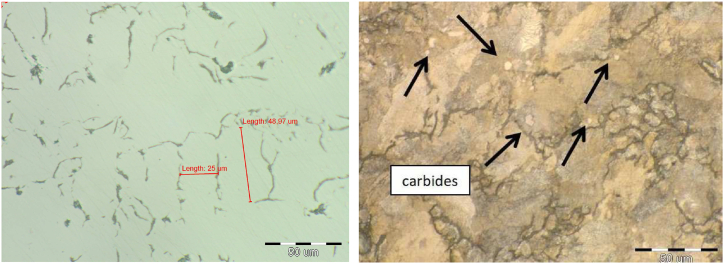
Microstructure of melt No. 5.

The microstructure of all melts was pearlitic with 92–96 % portion of pearlite, Fig. 1 – melt No 1., Fig. 2 – metl No. 3. Cementit in the structure of melts was not observed. In melt No. 5 (synthetic grey iron) there was a fully pearlitic microstructure and carbides were detected, Fig. 3. The occurrence of carbides was the reason for the increased hardness in this grey iron alloy. Results of the metalografical analysis are shown in Table 3.

**Table 3:** Results of the metalografical analysis.

Melt No.	Perlite in structure [%]	Graphite Size [μm]	Allocation of lameral graphite
1.	92	60–120	Type C - mixed
2.	96	60–120	Type D – interdendritic undirected
3.	96	30–60	Type E − interdendritic undirected
4.	96	120–250	Type C – mixed
5.	100	60–250	Type C – mixed
6.	96	60–120	Type C - mixed

Based on this analysis, the following three melts were selected (melts 1, 3 and 6 from Table 1 and Table 2):

a) normal operating semi-synthetic cast iron,
b) melt with inoculant (FeSi75), temperature increase (1500 °C) and FeTi70 microalloying,
c) synthetic cast iron, inoculated with FeSi and FeTi70 micro alloying.

The portion of the charge material and the heat treatment temperature for the selected three melts are given in Table 4.

**Table 4:** The portion of the charge material and overheating temperature for selected melts.

Cast No.	Melt No.	Charge Material (wt. %)								Temperature [°C]
		Steel Scrap (Sheet Metal)	Return Material	PIG Iron	FeSi75	FeMn80	Carburizer (Desulco 9001)	Inoculant (Added to the Iron Stream)	FeTi70	
A	1.	32.8	53.3	10	–	0.4	1.3	0.20	–	1420
B	3.	35.5	52.9	9.8	0.12	0.5	0.98	0.20	0.28^a^	1500
C	6.	97.5	–	–	0.60	0.47	0.67	0.50	0.26^a^	1520

a FeTi70 – into the furnace with charge.

The aim of the presented study was to compare:

- levels of residual stresses,
- hardness HB.

**Fig. 4:**
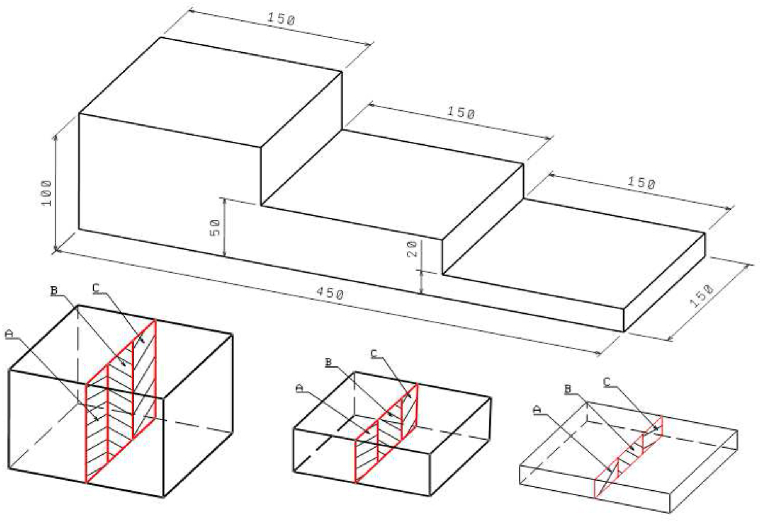
R-block (stairs) with indicated areas of HB hardness measurement [20].

The proposed methodology aimed to reduce the residual stress levels in the synthetic cast iron while maintaining the required hardness. The results of this research can be validated to some extent with data obtained in another research carried out on castings from the same melts, Table 2. Specifically, the HB hardness values measured on samples taken directly from the stress grid (casting) were compared with the hardness values measured by the "stairs" method (R-block), Fig. 4 [20]. The locations of the hardness measurements by the “stairs” method are marked in red.

**Fig. 5:**
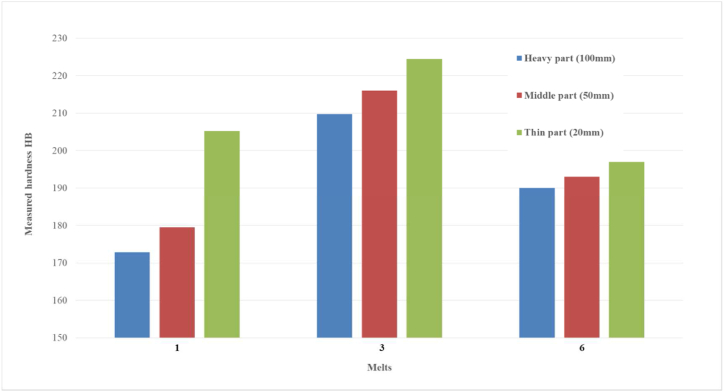
Measured Hardness HB on R-block (stairs test).

As already mentioned, hardness is important in terms of further machining of the castings. Fig. 5 is a graphical representation of the measured hardness HB values determined on castings from analysed melts 1, 3, and 6 (see Table 3) as a function of casting thickness (thin 20 mm; medium 50 mm and thick 100 mm).

## 3 Measurement of residual stresses

Experimental methods for determining residual stresses are divided into three basic groups according to the degree of damage to the test specimen: non-destructive; semi-destructive and destructive. For the determination of residual stresses in castings, the cutting method, which belongs to the destructive methods, was used.

**Fig. 6:**
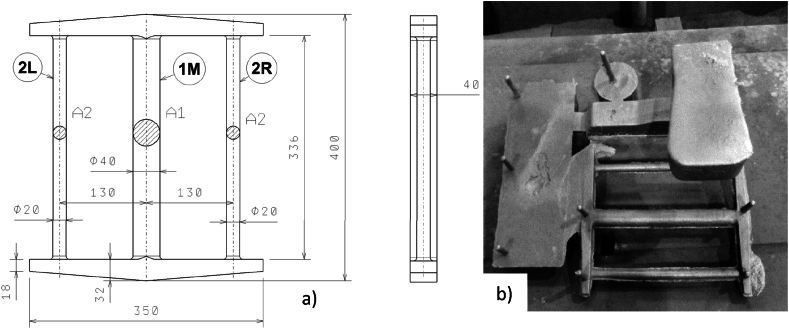
a) The basic dimensions and shape of the stress grid according to Sipp; b) view of the casting with the gating system.

In the present paper, measurements have been carried out on castings with a stress grid according to Sipp [16,21]. Its shape and dimensions can be seen in Fig. 6. The principle of the measurement consists of cutting the solid (thicker) bar and then evaluating the dimensional changes of the casting that occur due to the presence of residual stresses.

The residual stresses arise due to the higher cooling and shrinkage rate of the solid bar (thicker - marked 1 M) in the temperature region of elastic deformation compared to the thinner bars (marked 2L and 2R), which are almost cooled at that time, preventing the solid bar from shrinking. After the casting has cooled, tensile and compressive residual stresses are induced in the solid bar (1 M) and thin bars (2L, 2R), respectively. Based on the data obtained, the propensity of the alloy to the formation residual stresses can be assessed. The Sipp test is based on the conditions of static equilibrium in the volume of the lattice according to relation (1):

(1) $$\sigma_{1}∙A_{1}={2\;\sigma }_{2}∙A_{2}$$

where $\sigma_{1}$ is the uniaxial tensile stress in the solid bar 1 M,

$\sigma_{2}$ - uniaxial compressive stress in the thin bar (2L, 2R),

$A_{1}$ - cross-sectional area of the solid bar,

$A_{2}$ - cross-sectional area of the thin bar.

The magnitude of the normal stresses in the solid and thin bar investigated on the Sipp stress grid can be written in the following form, taking into account Hooke's law:

(2) $${+\sigma }_{1}=\varepsilon_{1}∙E$$

(3) $${-\sigma }_{2}=\varepsilon_{2}∙E$$

where $\varepsilon_{1}$ is the strain in the solid bar

$\varepsilon_{2}$ – strain in the thin bar

E – Young's modulus of elasticity of the casting material.

The dimensions in Fig. 6 show that the cross-sectional area of the more massive bar is 4 times larger than that of the thinner bar. Based on the above fact, by modifying equations (1), (2), (3), we obtain (4):

(4) $$\varepsilon_{2}={2\;\varepsilon }_{1}$$

Based on equation (4), it can be concluded that there is a linear dependence between the principal strain $\varepsilon_{1}$ in the solid and the principal strain $\varepsilon_{2}$ in the thin bar in the elastic deformation region. The authors of this paper decided to use the strain gauge method to measure the strains, which allows to register directly the proportional strains on the surface of the specimen at the measured location. The advantage of the chosen method is that the strain gauge sensor can be placed at any location on the surface of the casting under analysis.

The Sipp stress grid is designed to produce uniaxial stress in three parallel bars. The standard procedure for measuring tensile/compressive stress, excluding bending and torsion, is a half-bridge connection of two strain gauges located in the same cross-section on opposite sides. The advantage of this configuration is that it allows to evaluate the value corresponding to uniaxial stress only. Due to the fact that in this case, the measurements are on castings, where thermal processes play a large role, the authors decided to use a quarter-bridge configuration. In the case where only uniaxial stresses are applied, the measured values at two opposite locations in the same cross-section should be approximately the same. The advantage of the quarter-bridge connection is that the measured values can provide very important information about the presence of, for example, a bending effect. The above procedure later proved to be correct, as experimental measurements also identified bending effects caused by the gradual cooling of the casting.

**Fig. 7:**
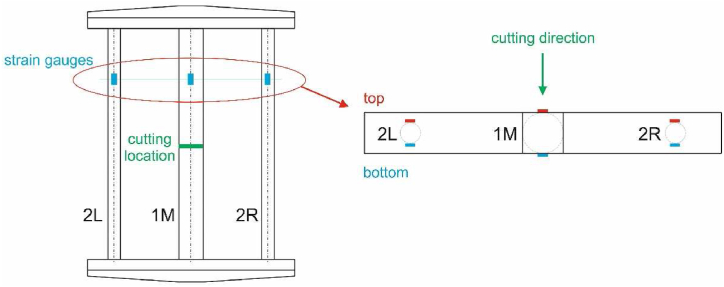
Locations of the strain gauges on the casting.

A schematic of the specimen with the location of the strain gauges for the experimental measurement of residual stresses is shown in Fig. 7. The locations of the strain gauges were chosen in the same cross-sections as seen in the figure (marked in blue). The effect of temperature change during the residual stresses measurement can be neglected because the room temperature was constant and the strain gauges were applied at a sufficient distance from the cutting location.

**Fig. 8:**
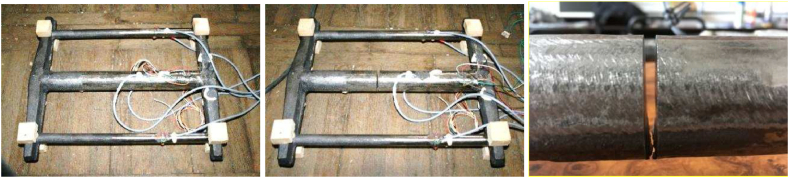
View of the cast with applied strain gauges a) before cutting; b) after cutting; c) detail in the place of cutting.

Strain gauges type 1-LY11-10/120 (HBM, Germany), Quantum MX840 measuring apparatus (HBM, Germany), and CatmanAP evaluation software (HBM, Germany) were used for the measurements. As mentioned before, the residual stresses were evaluated based on the measured strains registered by the strain gauges. Fig. 8 shows a view of the casting with strain gauges applied before (Fig. 8a) and after cutting (Fig. 8b). The casting was positioned so that the inlet system was on the top side during cutting.

**Fig. 9:**
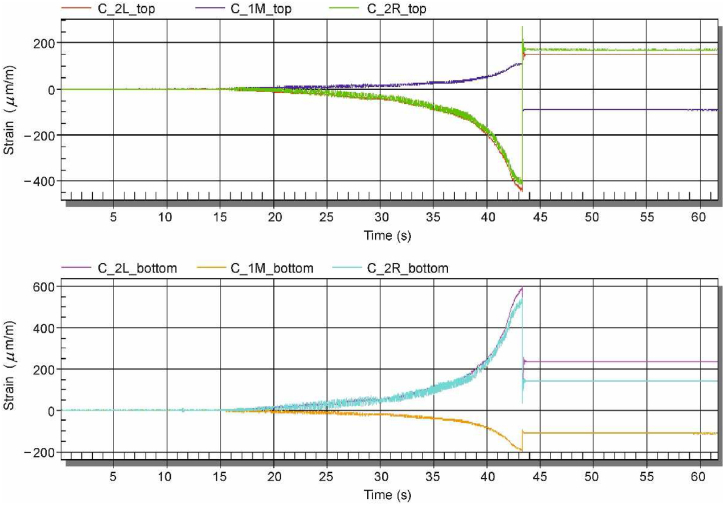
Time recording of measured strains during cutting of the casting.

In Fig. 9, the time record of the strains registered during the cutting process is documented. The increase or decrease in the measured strains was caused by the clamping effect from the cutting tool. After the bar was cut, there was a step change in the strains, at about 43 s, when the measured data stabilized.

Table 5 shows the measured values of the strains read at the end of the measurement. Since the measured values were obtained in a quarter-bridge connection, they take into account not only the effect of the uniaxial strain on the bars but also the bending effect due to the cooling of the casting.

**Table 5:** Measured strains in individual rods after cutting the castings.

Cast No.	Position	Measured value of strains after cutting the casting (με)		
		Thin rod – 2L	Thick rod – 1 M	Thin rod – 2R
A (Melt 1, without Ti)	Top	261	−127	213
	Bottom	335	−176	304
B (Melt 3, with Ti)	Top	145	−79	139
	Bottom	220	−112	271
C (Melt 6, with Ti)	Top	155	−82	177
	Bottom	230	−105	143

Since the data were read at the end of the measurement (after the casting was cut), it can be concluded that the different values at the top and bottom of the bars are due to the temperature effect in the cooling process of the casting. From the measured data in the upper and lower fibres in the same cross-section when considering tensile and bending stresses, only the axial stress component can be determined from the stress distribution over the height of the cross-section, see Table 6. The measured positive value means that after the casting was cut, the bar which was compressed before cutting, elongated. That is, a positive value of the measured strains corresponds to compressive residual stresses and, conversely, a negative value corresponds to tensile residual stresses.

**Table 6:** Recalculated values of strains in cast rods excluding bending.

Cast No.	Measured value of strains after cutting the casting (με)		
	Thin rod – 2L	Thick rod – 1 M	Thin rod – 2R
A (Melt 1, without Ti)	298.0	−151.5	258.5
B (Melt 3, with Ti)	182.5	−95.5	205.0
C (Melt 6, with Ti)	192.5	−93.5	160.0

According to Sipp, residual stresses in castings are evaluated by comparing the elongation of the bars after the casting has been cut. The bar elongation is directly related to the proportional deformation, therefore the strain gauge method chosen by the authors can be considered appropriate. The analysis of the experimentally measured data documents, among other things, that during casting there is a different stress distribution along the height of the casting due to temperature effects, which can also be considered as a valuable finding.

**Fig. 10:**
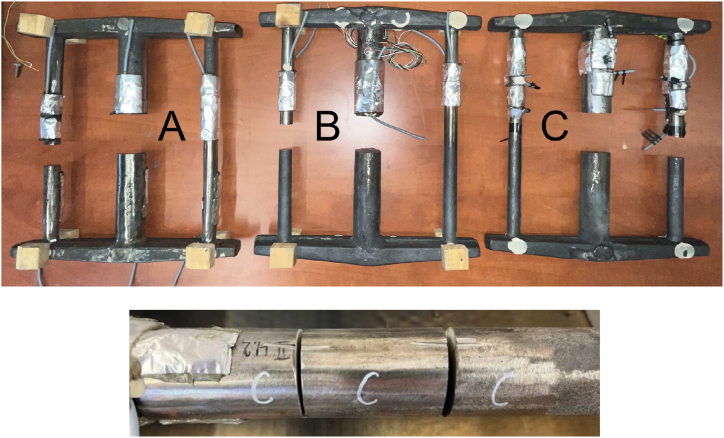
Taking samples for hardness measurement from analysed castings.

In addition to residual stresses, the authors evaluated hardness in both thicker and thinner bars on the castings analysed. A view of the castings with the sampling locations is shown in Fig. 10. The aim was to compare the measured hardness values for castings from the same melt but with those obtained by the stairs method [20]. The authors expected slight deviations since in the stairs method the locations measured were on a smooth surface (Fig. 1) with thicknesses of 20 mm and 50 mm. For the castings presented, the locations measured were at the centre of the circular cross-sections, that is, 20 mm from the edge for the thicker bar and 10 mm from the edge for the thinner bar, Fig. 10. The measurement was made using a hardness tester HPO 3000 (conditions: 10/3000/10), the same conditions as in the foundry.

Table 7 compares the hardness values according to the stairs method and on samples taken from the castings analysed.

**Table 7:** Measured hardness values according to the stairs method and on samples taken from the castings analysed.

Cast No.	Steel scrap (sheet metal) (%)	Ti (%)	HB [20] thick	HB [20] thin	HB on Sipp rod thick rod	HB on Sipp rod thin rod
A (Melt 1, without Ti)	32.8	–	186	205	199	252
B (Melt 3, with Ti)	35.5	0.183	217	224	217	310
C (Melt 6, with Ti)	97.5	0.192	197	209	202	257

## 4 Conclusion

Based on the experimentally obtained data, it can be concluded:

- In the samples with added Ti (No. 3 and No. 6), there was a significant decrease in the residual stress levels compared to the casting without added Ti (No. 1), see Table 6. Microalloying with FeTi positively affects the sensitivity of the dispersion of the mechanical properties of cast iron due to the change in the wall thickness of the casting, as well as the reduction of residual stresses. In samples 3 and 6, there was a reduction in residual stress levels. In synthetic cast iron (melt No. 5), carbides were observed in the structure (Fig. 3). By microalloying Ti, their presence in the structure of synthetic cast iron (melt No. 6) was not confirmed, which also has a positive effect on residual stresses.
- By comparing castings with added Ti made from pig iron and steel scrap (synthetic cast iron), lower levels of residual stresses were measured on the synthetic alloy.
- Based on the measured values in the upper and lower fibres for all three castings, it can be concluded that there was a non-uniform distribution of stresses over the height of the casting, Table 4. This is most likely related to the thermal cooling process of the casting.
- The measured hardness HB values showed good agreement with the stairs method. For thinner bars, the measured values were more than 20 % higher against the stairs method, since the cross-sections were thinner.

The experimentally determined values confirmed the theoretical assumptions of how a decrease in residual stresses can be achieved for synthetic alloys (steel scrap with added Ti) in comparison to pig iron alloys.

Currently, the price of steel scrap is in the range of 400–450 €/tons and the price of pig iron is 600–700 €/tons. This is not only an economic saving compared to the normal production of cast iron from pig iron (almost 40 %), but in addition, the processing of scrap steel results in an energy reduction in terms of raw material input [22,23].

The implementation of synthetic cast iron production in an already established foundry is time and economically acceptable and there is a possibility to return to pig iron production in case of unavailability of scrap steel.

## Data availability

The datasets used and/or analysed during the current study available from the corresponding author on reasonable request.

## CRediT authorship contribution statement

**Peter Futas:** Writing – original draft, Funding acquisition, Data curation, Conceptualization. **Miroslav Pástor:** Writing – review & editing, Validation, Software, Methodology, Data curation, Conceptualization. **Alena Pribulova:** Writing – review & editing, Resources, Project administration, Investigation.

## Declaration of competing interest

The authors declare that they have no known competing financial interests or personal relationships that could have appeared to influence the work reported in this paper.
